# Intensified low-density lipoprotein-cholesterol target of statin therapy and cancer risk: a meta-analysis

**DOI:** 10.1186/s12944-015-0147-6

**Published:** 2015-11-02

**Authors:** Haixia Sun, Yang Yuan, Pin Wang, Rongrong Cai, Wenqing Xia, Rong Huang, Shaohua Wang

**Affiliations:** Department of Endocrinology, The Affiliated ZhongDa Hospital of Southeast University, No.87 DingJiaQiao Road, Nanjing, 210009 PR China

**Keywords:** Intensified LDL-c target of statin therapy, Cancer, LDL-c

## Abstract

**Background:**

This study aimed to investigate the relationship between an intensified low-density lipoprotein-cholesterol (LDL-c) target of statin therapy and cancer risk.

**Methods:**

Data from PUBMED, EMBASE, and the Cochrane Central Register of Controlled Trials as of September 2014 were searched for randomized controlled trials on statins. An intensified LDL-c target of <2.59 mmol/L (100 mg/dL) or a relative LDL-c reduction by at least 30 % of the baseline was the primary criterion for all the trials that were included in this meta-analysis. The *I*^2^ statistic was used to measure heterogeneity among the trials, and risk estimates were calculated for cancer incidence in this random-effect meta-analysis.

**Results:**

Nine eligible studies were identified with 59,571 participants, of whom 5379 developed cancer during the follow-up period (2691 were given statins and 2688 were given control treatment). The intensified LDL-c target of statin therapy did not affect cancer incidence (odds ratio, 1.00; 95 % confidence interval, 0.94 − 1.06; *I*^*2*^ = 1.6 %, *p* = 0.42), which included some common cancers. Subgroup analysis showed that neither the chemical properties nor the variety of the statins accounted for the residual variation in risk.

**Conclusions:**

The intensified LDL-c target of statin therapy had no effect on the overall incidence of cancer, including some common cancers. Therefore, intensified statin therapy does not need to be changed among adult clinical patients.

## Introduction

Statin therapy has been proven to help prevent cardiovascular events and mortality [1–6]. Intensified low-density lipoprotein-cholesterol (LDL-c) targets of statin therapy, which are highly accurate, have also been widely used in clinical practice, especially among high-risk patients. The third report of an expert panel on the detection, evaluation, and treatment of high blood cholesterol in adults (Adult Treatment Panel ATP III, 2001) recommended a LDL-c goal of < 100 mg/dL for patients with coronary heart disease (CHD) and CHD risk equivalents (10-year risk > 20 %; Framingham risk score) [7]. Among diabetic patients, the dyslipidemia control criteria recommend LDL-c < 2.59 mmol/L for diabetic patients and LDL-c < 2.07 mmol/L (80 mg/dL) for diabetic patients with cardiovascular disease in China [8, 9]. The American Association of Clinical Endocrinologists 2012 guidelines recommend LDL-c < 100 mg/dL for high-risk patients (≥2 major risk factors and Framingham risk score > 20 % CHD risk equivalent) [10]. The intensified LDL-c target of statin therapy in this meta-analysis was defined as a target of LDL-c < 2.59 mmol/L (100 mg/dL) or a relative LDL-c reduction of at least 30 % of the baseline [11].

Nevertheless, some side effects of statin therapy and the intensified LDL-c target of statin therapy have been reported (e.g., cancer risk). Several studies have reported the relationship between statin therapy and cancer risk [12–18]. A considerable amount of scholars believe that statin therapy has no relationship with cancer risk or statin therapy may prevent cancer, despite contradictory findings in the literature [15–18]. For instance, a significant decrease in HR-negative breast cancer was reported among statin users in a previous study [15]. A recently published systematic review and meta-analysis reported that statins have no effect on the overall incidence of cancer [17]. Few studies investigated the correlation between the intensified LDL-c target of statin therapy and cancer risk, but the same indefinite conclusion was obtained. Low risk of cancer was found for patients treated with high-efficacy statins in a recent population-based study (LDL-c reduction: low, ≤30 %; moderate, 31–40 %; high, ≥41 %) [19, 20]. However, a previous study showed that cancer incidence did not increase in the group achieving LDL-C < 30 mg/dL after high-intensity statin therapy compared with the controls [21].

The relationship between the intensified LDL-c target of statin therapy and cancer risk has been investigated for a long time, but results remain inconsistent. Thus, we aimed to investigate whether the intensified LDL-c target of statin therapy contributes to the onset of carcinoma according to the findings of previous meta-analyses and recent randomized controlled trials (RCTs).

## Methods

### Ethics

The protocol of the current study was approved by the research ethics committees of ZhongDa Hospital, which was affiliated with Southeast University.

### Search strategy

To obtain all of the original studies on the effect of intensified LDL-c target of statin therapy on cancer incidence in adult patients, we searched potentially eligible studies in the electronic databases PUBMED, EMBASE, and Cochrane as of September 2014. The following medical subject headings and free text keywords were used: “hydroxymethylglutaryl coenzyme A reductase inhibitor,” “statins,” “statin,” “fluvastatin,” “mevastatin,” “compactin,” “pravastatin,” “simvastatin,” “lovastatin,” “pitavastatin,” “rosuvastatin,” “cerivastatin”, “atorvastatin,” “cancer,” “carcinoma,” “neoplasm,” “tumor,” “phyma,” “randomized controlled trial,” and “human”.

### Selection criteria

We included the RCTs if one of the endpoint or study assesses the effect of intensified LDL-c target of statin therapy on cancer endpoints in adult patients. The inclusion criteria used for the search are described as follows. The study should be an original work comparing statin treatment with an inactive control (placebo or no statins) involving more than 1000 adult study participants (18 years and older) whose cancer incidence was reported and followed-up for over one year. The patients included in the report should have achieved an intensified LDL-c target of < 2.59 mmol/L (100 mg/dl) or a relative LDL-c reduction of at least 30 % of the baseline. We excluded comparison trials involving either different statins or different doses of the same statin as well as trials on patients with cancer.

### Data sources

For all of the published trials, the following details were recorded: study characteristics (study design and allocation), participants (baseline age, sample size, and accompanying disease), therapeutic intervention (type of statin, dose of statin, and duration of therapy), and new cancer cases. Moreover, we recorded the endpoint LDL-c concentrations and relative reduction in LDL-c concentrations during the statin therapy to verify whether the intensified LDL-c target of statin therapy correlates with the incidence of cancer.

For trials with unpublished information, we formally requested data using a question sheet. The questions covered the number of incident cancer in specific sites (e.g., central nervous system (CNS), skin, breast, respiratory, gastrointestinal, and hematological). However, the replies were unclear, or no reply was received.

### Statistical analysis

To determine the potential effects of the intensified LDL-c target of statin therapy on the incidence of cancer, odds ratio (OR) and 95 % confidence interval (CI) were used to compare the mean differences in each subgroup separately. We evaluated the statistical heterogeneity between trials with the *I*^*2*^ statistic (with 95 % CIs), which is derived from Cochran’s Q (100 × (Q-df)/Q) [22] and provides a measure of the overall variation that is attributed to between-trial heterogeneity. Random-effect meta-analysis was performed instead of the fixed-effect model because the former approach provides a more conservative assessment (e.g., broad CIs) of the average effect size. Subgroup analyses were used to investigate the potential sources of heterogeneity between trials. The factors that were investigated included the type of statin, dose of statin, and relative reduction in LDL-c concentrations. We analyzed the data with Stata version 11.0.

A funnel plot and Egger’s test were used to test for publication bias [23]. We conducted meta-analyses that included all trials; different types of statin (simvastatin, rosuvastatin, atorvastatin, pravastatin, and fluvastatin); comparison trials with hydrophilic (pravastatin and rosuvastatin) and lipophilic (atorvastatin, simvastatin, and fluvastatin) statins; trials with specific cancer according to available date (respiratory, gastrointestinal, genitourinary and CNS). Additionally, we performed the sensitivity analysis with Stata version 11.0.

## Results

### Description of the studies

**Fig. 1:**
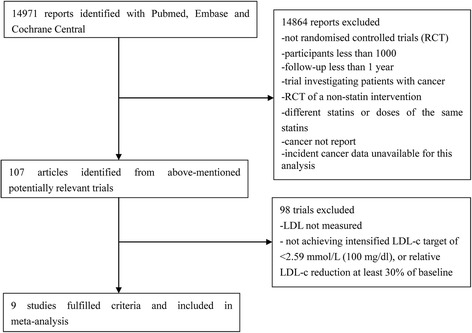
Flow diagram of literature search to identify randomised placebo-controlled or standard care-controlled statin trials

We identified nine trials involving 59,571 non-cancer participants, of whom 5379 developed cancer (Fig. 1). Five different statins were studied, with a follow-up duration of 1.9–16.3 years. The characteristics of the included studies are summarized in Tables 1 and 2.

**Table 1:** Characters for non-cancer participants in nine placebo-controlled and standard care-controlled statin trials

Study	Year	Number of participants	Type	Mean follow-up (years)	Age (years)	Statin	Jadad Score
HPS	2011	20536	At increased risk of vascular events	16.3	40–80	simvastatin 40 mg	5
AURORA	2009	2773	With maintenance hemodialysis	3.2	50–80	rosuvastatin 10 mg	5
CARDS	2008	2838	With type 2 diabetes and no history of coronary heart disease (CHD)	3.9	40–75	atorvastatin 10 mg	4
JUPITER	2008	17802	Healthy	1.9	50 years and older	rosuvastatin 20 mg	6
GDDS	2005	1255	With type 2 diabetes receiving maintenance hemodialysis	4	18–80	atorvastatin 20 mg	7
ALLIANCE	2004	2442	CHD patients with hyperlipidemia	4.3	61.1(atorvastotin)/61.3(usual-care)	a maximum atorvastatin dose of 80 mg/day	3
PROSPER	2002	5804	With a history of, or risk factors for, vascular disease	3.2	70–82	pravastatin 40 mg	6
LIPS	2002	1677	After successful first percutaneous coronary intervention (PCI)	3.9	18–80	fluvastatin 80 mg	6
4S	1994	4444	With CHD	5.4	35–70	simvastatin 20 mg	5

**Table 2:** Characters for non-cancer participants in nine placebo-controlled and standard care-controlled statin trials

Study	Cancer outcome	Relative LDL-c reduction	endpoint LDL-c (mmol/L)	New cancer cases	Number in statin group	Number in control group	New cancer in satin group	New cancer in control group
HPS	CNS, Gastrointestinal, genitourinary, respiratory et al.	32.35 %	2.3	3493	10269	10267	1749	1744
AURORA	Not specified	43 %	1.1	225	1389	1384	107	118
CARDS	Not specified	below 2.59 mmol/L	1.99	141	1428	1410	69	72
JUPITER	Not specified	49 %	1.42	612	8901	8901	298	314
GDDS	Not specified	42 %	1.86	83	619	636	39	44
ALLIANCE	Not specified	34.30 %	2.5	144	1217	1225	67	77
PROSPER	Gastrointestinal, genitourinary, respiratory et al.	34 %	2.5	444	2891	2913	245	199
LIPS	Gastrointestinal, genitourinary, respiratory, CNS et al.	below 2.59 mmol/L	2.48	32	844	833	14	18
4S	Gastrointestinal	35 %	3.17	205	2221	2223	103	102
TOTAL				5379	29779	29792	2691	2688

### Effects of the intensified LDL-c target of statin therapy on the overall incidence of cancer

#### Any statin

**Fig. 2:**
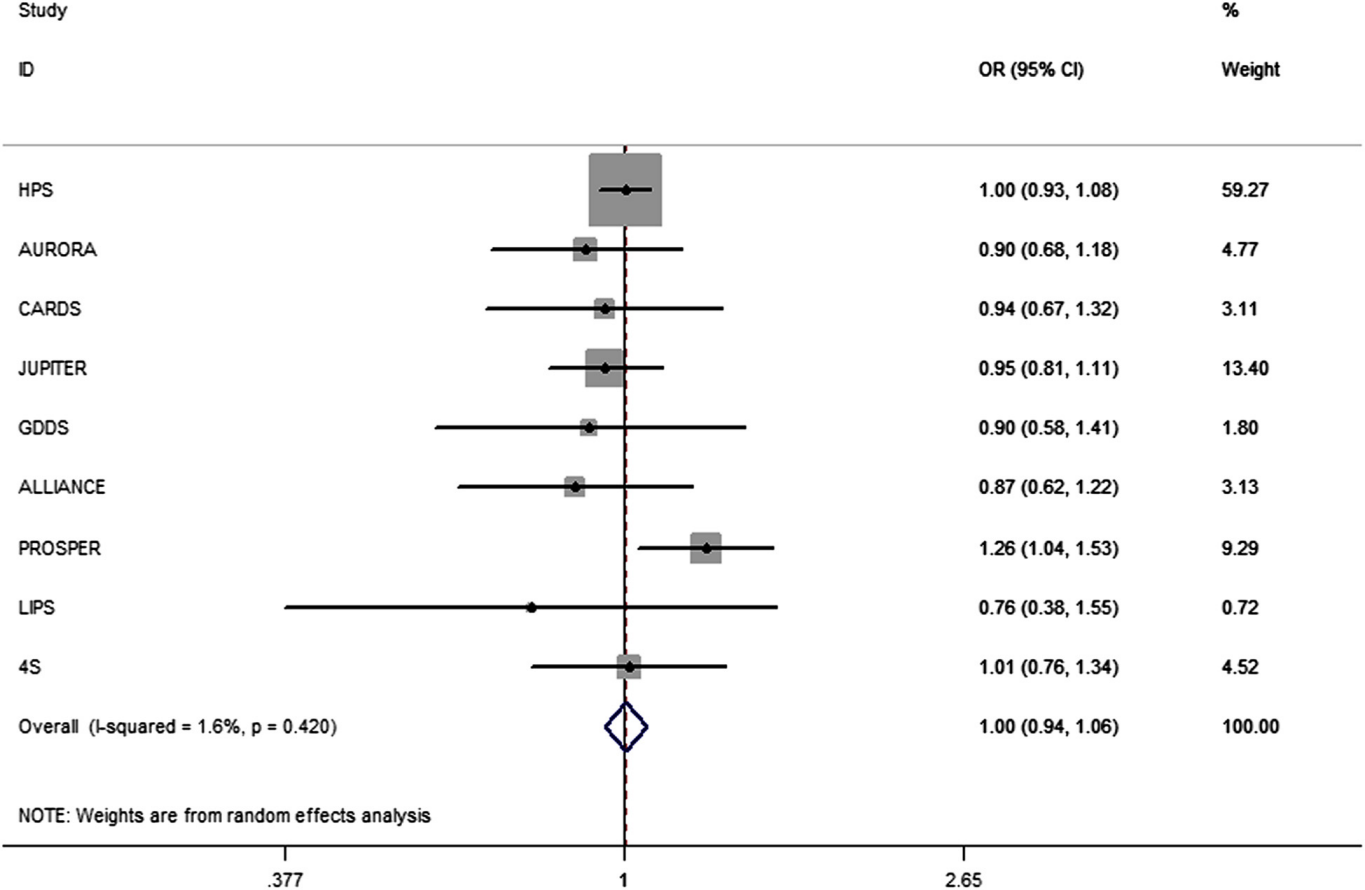
Association between intensified LDL-c target of statin therapy and incident cancer in 9 major trials. Legend: The intensified LDL-c target of statin therapy has no effect on the overall incidence of cancer

The intensified LDL-c target of statin therapy did not affect the overall incidence of cancer (OR, 1.00; 95 % CI, 0.94 − 1.06; *I*^*2*^ = 1.6 %, *p* = 0.42; Fig. 2). Among the nine trials, only the PROSPER (pravastatin in elderly individuals at risk of vascular disease) study [13] showed that the intensified LDL-c target of pravastatin therapy promoted cancer incidence (Fig. 2).

We also performed sensitivity analysis, which suggested that the combination of all trials in the primary analysis was appropriate. Additionally, we conducted a funnel plot and Egger’s test for all nine trials and found no underlying publication bias.

### Different statins

**Fig. 3:**
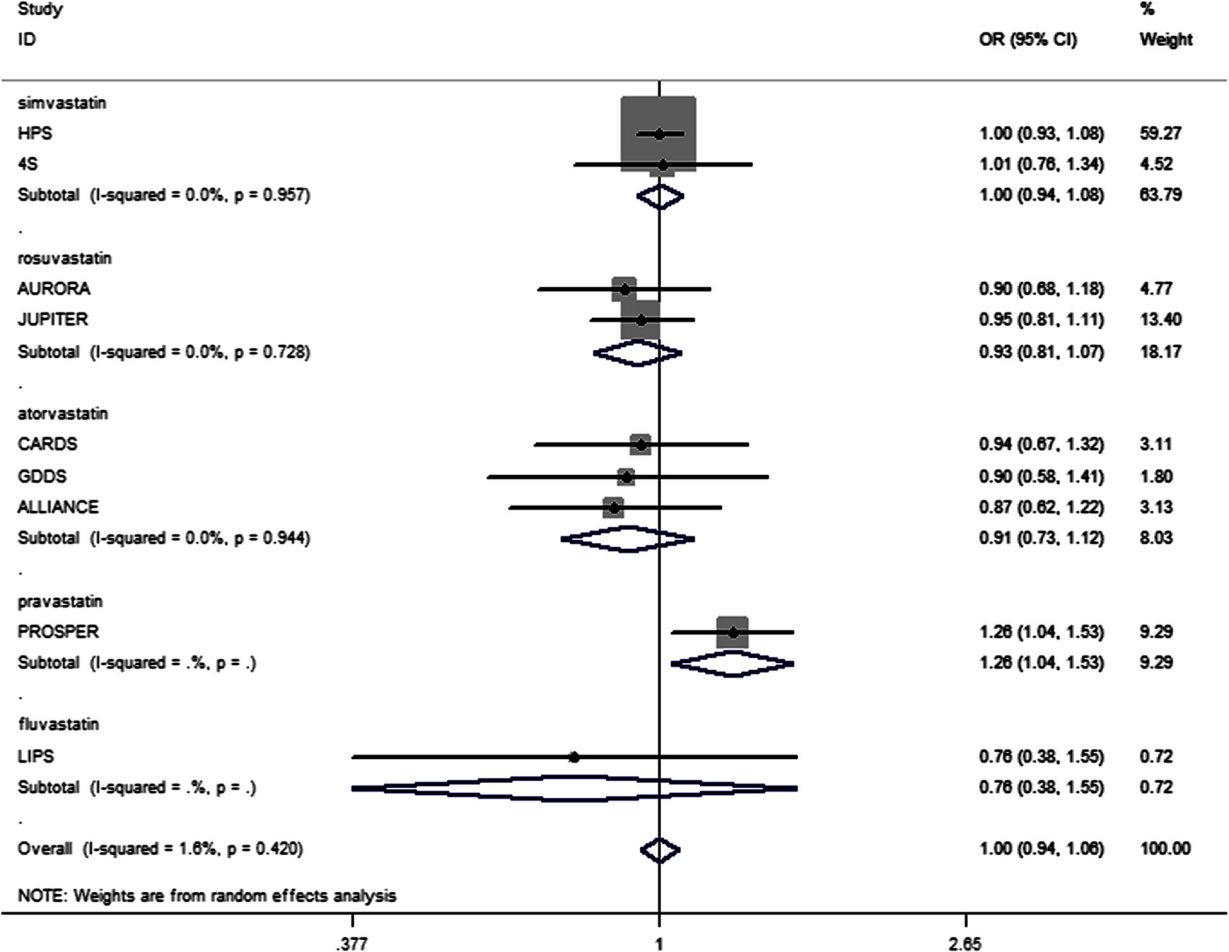
Association between different statins met intensified LDL-c target goal and incident cancer. Legend: The subgroup analyses of the individual statin that met the intensified LDL-c target showed no significant effect on the incidence of cancer. Pravastatin therapy seemed to have favorable promoting effect on cancer incidence. The subgroup analyses of fluvastatin, simvastatin, rosuvastatin and atorvastatin therapy showed no effect on cancer incidence

The subgroup analyses of the individual statin that met the intensified LDL-c target revealed no significant effect on the incidence of cancer. Pravastatin therapy was found to promote cancer incidence (OR, 1.26; 95 % CI, 1.04 − 1.53). The subgroup analyses of fluvastatin therapy [24] showed no effects on cancer incidence (OR, 0.76; 95 % CI, 0.38 − 1.55). This result (Fig. 3) was similar to that of simvastatin therapy (OR, 1.00; 95 % CI, 0.94–1.08; *I*^*2*^ = 0.0 %, *p* = 0.957) [25, 26], rosuvastatin therapy (OR, 0.93; 95 % CI, 0.81 − 1.07; *I*^*2*^ = 0.0 %, *p* = 0.728) [6, 27], and atorvastatin therapy (OR, 0.91; 95 % CI, 0.73 − 1.12; *I*^*2*^ = 0.0 %, *p* = 0.944) [28–30].

### Lipophilic and hydrophilic statins

**Fig. 4:**
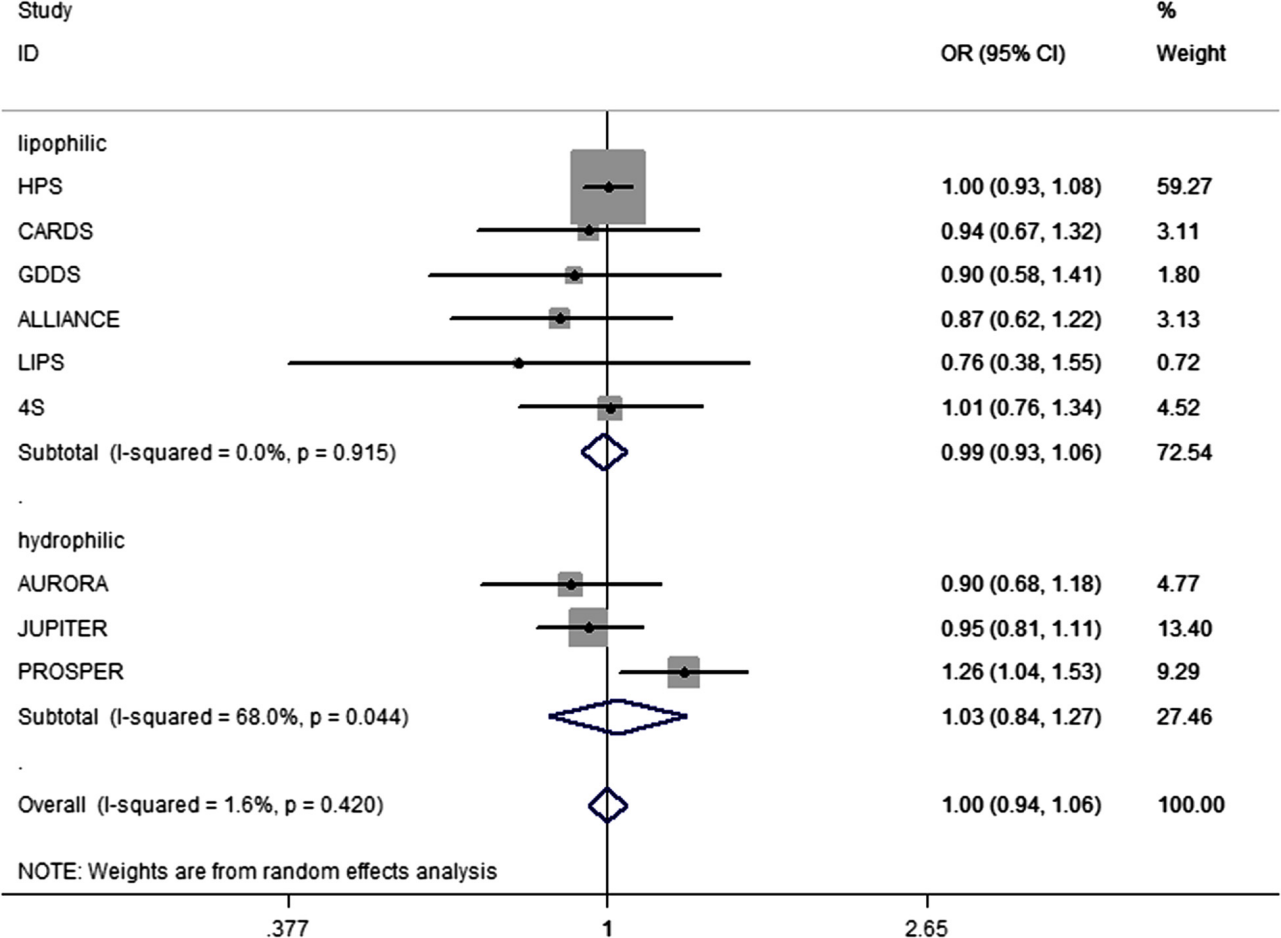
Lipophilic and hydrophilic statins and incident cancer. Legend: Neither the lipophilic nor hydrophilic statins that met the intensified LDL-c target had effect on the incidence of cancer

Given that the chemical properties of statins may lead to different results from our previous findings, statins were divided into two groups, namely, the lipophilic and hydrophilic statins, in another subgroup analysis. Neither the lipophilic (OR, 0.99; 95 % CI, 0.93 − 1.06; Fig. 4). nor hydrophilic (OR, 1.03; 95 % CI, 0.84 − 1.27; Fig. 4). statins that met the intensified LDL-c target affected the incidence of cancer.

### Effects of the intensified LDL-c target of statin therapy on cancer at specific sites

We also conducted subgroup analyses on site-specific cancer. However, the available data are limited. We only performed subgroup analyses on respiratory, CNS, gastrointestinal, and genitourinary cancers based on the present data. No relationship was found between the intensified LDL-c target of statin therapy and the incidence of respiratory cancer (OR, 1.05; 95 % CI, 0.91 − 1.21; *I*^*2*^ = 0.0 %, *p* = 0.794), CNS cancer (OR, 0.71; 95 % CI, 0.09–5.55; *I*^*2*^ = 55.5 %, *p* = 0.134), gastrointestinal cancer (OR, 1.11; 95 % CI, 0.80 − 1.54, *I*^*2*^ = 54.2 %, *p* = 0.088), and genitourinary cancer (OR, 1.02; 95 % CI, 0.91 − 1.14; *I*^*2*^ = 0.0 %, *p* = 0.986).

## Discussion

The intensified LDL-c target of statin therapy, with the goal of achieving LDL-c < 2.59 mmol/L (100 mg/dl) or a relative LDL-c reduction by at least 30 % of the baseline after statin treatment, is commonly used in clinical trials and is believed to be effective. The intensified LDL-c target of statin therapy is suitable for the general population. For example, according to previous reports, all patients with peripheral arterial disease or those with familial hypercholesterolemia benefit from the intensified LDL-c target of statin therapy [31, 32]. Furthermore, this approach is appropriate and widely used in high-risk populations, such as diabetics with cardiovascular and cerebrovascular diseases. These patients are known to be at high risk of cancer incidence, so lipid control appears worthwhile. The dyslipidemia control criteria recommend LDL-c < 2.59 mmol/L for diabetic patients, and LDL-c < 2.07 mmol/L (80 mg/dL) for diabetic patients with cardiovascular disease [8, 9]. Numerous studies have explored the relationship between statins and cancer incidence. However, reports on the effect of the intensified LDL-c target of statin therapy on the incidence of cancer are relatively few. The present comprehensive meta-analysis was performed to provide evidence to clinicians and patients, which can also improve patient compliance.

Our meta-analysis showed that the intensified LDL-c target of statin therapy had no effect on the overall incidence of cancer, including some common cancers. The results of this meta-analysis were relatively stable according to sensitivity analysis. Begg’s funnel plot and Egger’s test showed no underlying publication bias.

In the studies selected for our meta-analysis, PROSPER [13] indicated that pravastatin may increase cancer risk. However, when the authors of the PROSPER [13] study extended their follow-up period to 14 years, no relationship was found between statin use and cancer risk [33]. Another study showed that pravastatin therapy presented an increasing risk of cancer incidence with rising patient age [34]. Nevertheless, the findings on pravastatin need to be confirmed by further studies. To date, clinical statin therapy has no special significance according to our subgroup analysis.

Previous studies have shown that statin therapy may inhibit cancer. For example, a recent trial revealed that a cumulative amount of statin use may decrease prostatic cancer risk [35]. In a recently published study, lipophilic statin was reported to play a therapeutic role in cancer treatment [36]. Statins may inhibit HMG-CoA reductase to lower the concentration of mevalonate, thereby decreasing the amount of isoprenylated intermediates that are known to affect signaling pathways, from cancer formation to progression [37].

Given the confusing association between statin therapy and cancer risk, as well as increasing incidence of cancer, we conducted subgroup analyses on site-specific cancer. The results showed the absence of a relationship between the intensified LDL-c target of statin therapy and the incidence of respiratory, CNS, gastrointestinal, and genitourinary cancers. To determine the safety of clinical statin treatment, we should investigate the relationship between the intensified LDL-c target of statin therapy and the risk of individual cancers in the future.

Several limitations of our study should be noted. First, the number of studies included in our meta-analysis is limited, especially for some individual statin. Second, sufficient evidence was unavailable to support or contradict the finding that pravastatin may promote cancer incidence. Third, available evidence from unpublished data on site-specific cancer is limited.

## Conclusion

The intensified LDL-c target of statin therapy did not affect the overall incidence of cancer, including some common cancers, among adult patients. Therefore, the intensified therapy does not need to be changed among adult patients in clinical applications.

## Abbreviations

LDL-c: Low-density lipoprotein-cholesterol
CHD: Coronary heart disease
RCT: Randomized controlled trial
CNS: Central nervous system
OR: Odds ratio
CI: Confidence interval
